# Light-Driven Flying Balloons Based on Hybrids of Carbon Nanotubes and Cellulose Nanofibers

**DOI:** 10.3390/ma15217739

**Published:** 2022-11-03

**Authors:** Takashi Ikuno, Kazuki Takahashi, Akari Kadogawa

**Affiliations:** Department of Applied Electronics, Graduate School of Advanced Engineering, Tokyo University of Science, Katsushika, Tokyo 125-8585, Japan

**Keywords:** carbon nanotubes, cellulose nanofibers, flying balloons

## Abstract

We have fabricated nanocarbon-based palm-sized cubic paper balloons that can be levitated by light irradiation. These paper balloons are composed of carbon nanotube (CNT) freestanding films and cellulose nanofiber (CNF) freestanding films. The number of CNT freestanding films (*N*_CNT_) and the number of CNF freestanding films (6-*N*_CNT_) among the six walls of the cube were varied. We investigated the effect of *N*_CNT_ on the levitation behaviors under light irradiation. We found that the balloons were levitated when *N*_CNT_ was greater than or equal to two. The levitation height was found to be increased by increasing *N*_CNT_.

## 1. Introduction

Indoor drones are expected to be used to monitor the sick and elderly in hospitals, observe the growth of crops in greenhouses, and inspect factories [1,2,3]. In order to fly in confined small indoor spaces where people and precision apparatus are present, it is necessary to realize small, lightweight, soundless, and windless flying objects. Therefore, we believe that small balloon-type flying objects are more suitable for this purpose rather than multi-rotor or fixed-wing flying objects [4,5]. We have developed the first flying balloons composed of carbon nanotube (CNT) freestanding films in the world [6]. Because CNTs have extraordinary physical properties, such as being lightweight, high heat resistance, and high electromagnetic wave absorbent [4,7,8,9,10,11,12], the air temperature inside the paper balloons composed of CNT freestanding films increases rapidly by light irradiation. As a result, the balloon can be levitated without any sound or wind.

In order to use our balloon as a monitoring device as described above, it is necessary to fabricate lightweight thin-film devices, such as sensors, on the balloon surface directly. For this purpose, there are some challenges that must be solved. First, it is necessary to replace some of the films on the balloon with insulator films, as these play the role of the substrate to the thin-film devices. All walls of the balloon reported previously were composed of CNT films. Because CNTs are electrical conductors, direct deposition of thin-film devices might result in electrical shorts. Second, because CNT balloons look like black-body objects [8], these have little design appeal. For use in indoor space, it is necessary to have an appearance with high design quality. To solve these problems, we replaced some of the films of the balloon with cellulose nanofiber (CNF) films because the CNF film is an electrical insulator and optically transparent. In addition, CNF has high mechanical strength and is a good gas sealant [13,14,15,16,17,18]. Thus, CNF films might be suitable materials for this application. However, there has been no report on the use of CNF freestanding film as a part of the light-driven flying balloon.

In this article, we report the fabrication of cubic paper balloons composed of both CNF and CNT films. Among the six walls of the cube, the number of CNT freestanding films (*N*_CNT_) and the number of CNF films (6-*N*_CNT_) were varied. Balloons were fabricated and their buoyancy characteristics were investigated while ensuring the bonding strength of each film. We also show the light-driven levitation properties as a function of the composition ratio of CNT/CNF films.

## 2. Materials and Methods

### 2.1. Fabrication of CNT Freestanding Films

A glass plate was cleaned with ethanol and acetone. Then, a multi-walled CNT (MWNT) film was deposited by a spray coating method on the glass substrate [6,19]. During the spraying, the substrate was heated at 150 °C by a hot plate in air. The solution used for the spraying was a mixture of MWNT ink (MW-I (2 wt%), Meijo Nano Carbon, Nagoya, Japan) and CNF aqueous solution (2G18-nanoforest-S-1LBC, 1 wt%, Chuetsu Pulp Industry, Toyama, Japan), at a mass ratio of 83:17. The typical diameter and length of the MWNT in the solution are approximately 10 nm to 40 nm and approximately 2 µm, respectively. After deposition, the MWNT freestanding film was obtained by peeling the MWNT film off from the glass substrate. The obtained film is a square with approximately 9 cm sides, and its film thickness and density are approximately 5.5 µm and 0.5 mg/cm^2^, respectively.

### 2.2. Fabrication of CNF Freestanding Films

A glass plate was cleaned with ethanol and placed on a hot plate heated to 110 °C in air. The glass substrate surface was sprayed with a 0.25 wt% CNF aqueous solution. After applying 45 mL of the aqueous CNF solution and allowing the water to evaporate, the CNF film was peeled off from the glass substrate using a special jig. The obtained film is a square with approximately 9 cm sides, and its film thickness and density are approximately 5.0 µm and 1.6 g/cm^3^, respectively.

### 2.3. Fabrication of CNT/CNF Hybrid Balloons

**Figure 1:**
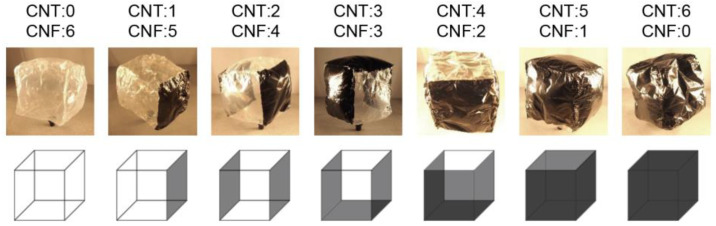
Photographs and illustrations of CNT/CNF hybrid balloons. The lengths of one side of the balloons were approximately 8 cm.

The obtained square films were patched together to form a development of regular hexahedral. When patching films together, a strong bond can be achieved by locally soaking the joint area with deionized water and allowing it to dry at room temperature (RT) of approximately 25 °C. Figure 1 shows photographs and illustrations of the fabricated balloons, in which the number of CNT planes and CNF planes was varied. The length of one side of the cube was approximately 8 cm. The weight of the balloon (total weight of the film and the mass of the air inside) was approximately 0.25 g.

### 2.4. Characterization

After the fabrication, the optical absorption property and the mechanical properties of the obtained freestanding films were characterized by an UV-vis-IR (JASCO, V-770, Tokyo, Japan) and a tensile tester (IMADA, ZTS-5N and MX2-500N, Aichi, Japan), respectively. The size of films used to characterize the mechanical properties were approximately 10 mm in width and 45 mm in length. The films were patched from two films (10 mm in width and 25 mm in length), with the patched length of approximately 5 mm. In addition, we also measured the temperature change, which is caused by light irradiation using tungsten lamps (300 W) as the light source and by the MWNT film using a thermography camera (AS ONE, TIM-03, Osaka, Japan). The temperature measurement was carried out at RT of approximately 25 °C.

**Figure 2:**
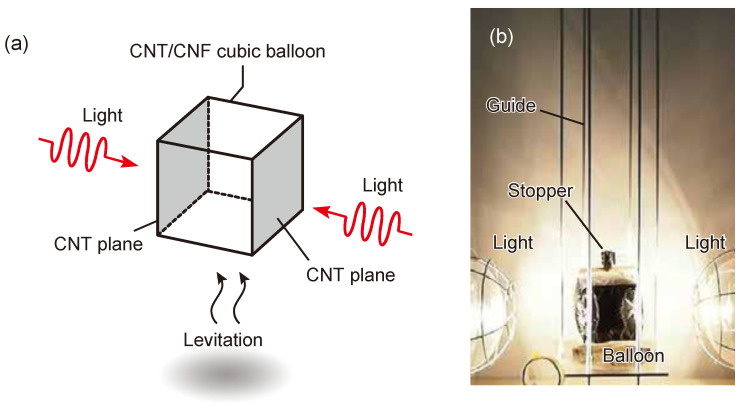
(**a**) Schematic illustration of a flying balloon based on CNT and CNF films. (**b**) Photograph of the experimental setup.

Figure 2a shows a schematic diagram of the characterization of balloon levitation. The balloon was irradiated with light from two tungsten lamps (300 W). The light irradiation causes buoyancy and balloon levitation. To measure the buoyancy, the lower part of the balloon was attached to an electronic balance, and the buoyancy was estimated from the change in mass. The buoyancy test was carried out using the experimental apparatus shown in Figure 2b. The balloon was housed in a guide consisting of four thin metal rods. A stopper suspended by a thread is placed at the top of the balloon. After 30 s of light irradiation, the stopper was removed, and the balloon was raised along the guide. The floating altitude was measured at this time.

## 3. Results and Discussion

**Figure 3:**
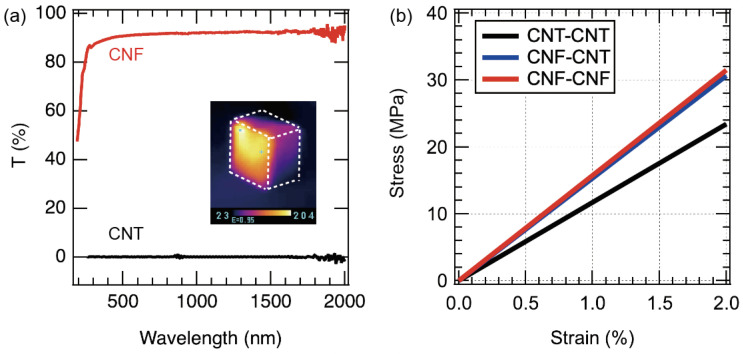
(**a**) Optical properties of CNF and CNT freestanding films. Inset is a thermograph of CNT balloon during light irradiation. (**b**) Mechanical properties of film-to-film junctions.

Figure 3a shows the transmittance of the obtained CNT and CNF films, where the CNT film has zero transmittance over the entire wavelength range and almost all the incident light is absorbed. On the other hand, the CNF film shows a transmittance of more than 85% at a wavelength of 300 nm, indicating that most of the incident light is transmitted in the wavelength range from the UV to the IR region. In other words, the CNF film absorbs almost no light, and therefore, the film is not heated by radiation heating. On the other hand, when the CNT film is irradiated with light, the temperature rises instantaneously. The inset shows a thermal image of a CNT balloon (all walls are CNTs) irradiated with a tungsten lamp from the left. It was found that the temperature immediately rose to more than 140 °C by light irradiation. On the other hand, it is easy to imagine that the internal temperature of the balloon rises slowly due to heat conduction and transfer from the surface film to the internal air.

When a balloon is irradiated with light, the temperature of the air inside the balloon rises, which increases the balloon pressure. It is difficult to know the mechanical properties of the film joints, which are considered to be the most mechanically weak part of the balloon. In this study, we investigated the bonding strength between CNT films, between CNF films, and between CNT films and CNF films. Five samples of each combination were prepared, and the stress-strain curves were measured. All curves were similar. Typical results are shown in Figure 3b. In the case of CNT films bonded to each other, the tensile strength was 53 MPa and Young’s modulus was 1.17 GPa. The tensile strength and Young’s modulus of CNF film bonded to each other were 146 MPa and 1.57 GPa, respectively. The tensile strength and Young’s modulus of the bonded CNT and CNF films were 108 MPa and 1.53 GPa, respectively. From these results, it was found that the strength was very high even if they were joined. Therefore, even if the internal pressure rises during light irradiation, there is little possibility that the joint will fail.

**Figure 4:**
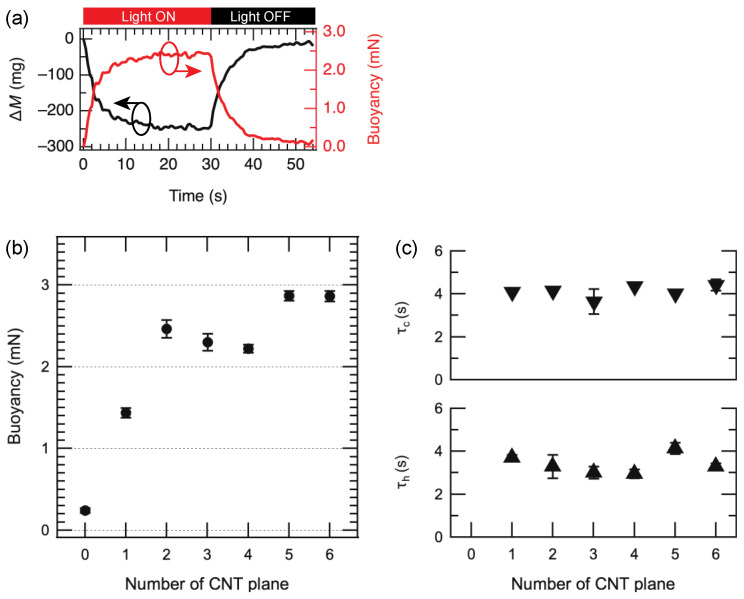
(**a**) Time evolutions of the mass change and buoyancy force of the balloon (number of CNT planes is 3) during the light irradiation. (**b**) Buoyancies of CNT/CNF hybrid balloons as a function of number of CNT planes. (**c**) Rise and decay time constant of the buoyancies as a function of number of CNT planes.

The buoyancy of each balloon was measured when it was exposed to light. Figure 4a shows the mass change Δ*M* of the balloon (*N*_CNT_ = 3) during the light irradiation and the experimental results of buoyancy force estimated from the mass change. The balloon was irradiated with light for 30 s. The mass change of the balloon decreased rapidly from the start of irradiation and became constant after approximately 20 s. The buoyancy force at this time was about 2.4 mN. The buoyancy forces were estimated against the number of CNT walls in the balloon, as shown in Figure 4b. It was found that the buoyancy force increased as the number of CNT walls increased, and saturation occurred when *N*_CNT_ was more than 2.

We estimated the time constant *τ*_h_ of the buoyancy change during light irradiation and *τ*_c_ of the buoyancy change when the light is off. These time constants were derived from the following equations:

$$F\left(t\right)\propto \left\{1-\exp\left(-\frac{t}{\tau_{h}}\right)\right\}\;(t\leq 30\;s)$$

$$F\left(t\right)\propto \exp\left(-\frac{t}{\tau_{c}}\right)\;(t>30\;s)$$

where *F*(*t*) and *t* are the buoyancy and time, respectively. *τ*_h_ and *τ*_c_ were plotted against *N*_CNT_ in Figure 4c. Both values are found to be independent of *N*_CNT_ and are almost constant. The time constants are considered to indirectly represent the time constant of the air temperature change such as the thermal conductivity of air. Although increasing *N*_CNT_ might increase the maximum temperature of the air, the time constant might be governed by the thermal conductivity of the air.

**Figure 5:**
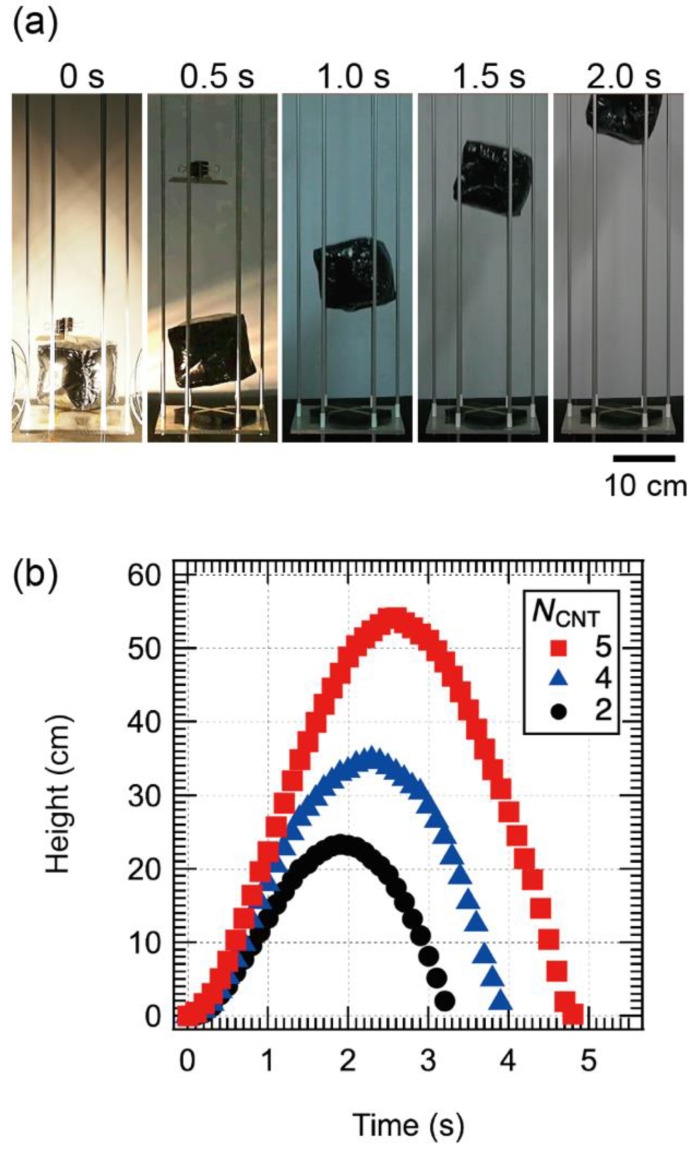
(**a**) Photograph of flying balloon (*N*_CNT_ = 5) after light irradiation for 30 s. (**b**) Time evolution of levitation heights of flying balloons as a function of number of CNT planes.

Next, we investigated the levitation characteristics of the CNT/CNF balloon. Figure 5a shows photographs of a CNT/CNF balloon with *N*_CNT_ = 5 during levitation. The left figure shows the snapshot when the stopper was released after 30 s of light irradiation with the stopper in place. The subsequent photographs of the balloon were taken every 0.5 s. We confirmed that the balloon rose about 48 cm in about 2 s.

Figure 5b shows the relationship between the floating altitude and the elapsed time after the release of the stopper for *N*_CNT_ = 2, 4, and 5. The highest altitude was about 54 cm for *N*_CNT_ = 5. The higher the *N*_CNT_, the higher the maximum altitude and the shorter the time to reach an arbitrary altitude. This may be due to the fact that the larger the *N*_CNT_, the higher the space temperature of the balloon and the greater the buoyancy, resulting in a faster and higher lift. It is interesting to note that the balloon was levitated even when the number of CNF walls was 4 (*N*_CNT_ = 2). Therefore, the CNT wall can be used as a heat source for levitation, and the other walls can be used for mounting electronic devices.

## 4. Conclusions

We have fabricated cubic paper balloons that levitate when exposed to light. The paper balloon was fabricated by patching CNT freestanding films and CNF freestanding films. We investigated the effect of *N*_CNT_ on the floating properties. When two or more of the six walls of the cube were CNT freestanding films, this cube was found to levitate upon light irradiation. Since the remaining four or less walls are insulating CNF freestanding films, electronic devices can be mounted on the CNF films of the cube. Various electronic devices have been reported to be fabricated on CNF films [13,20,21,22,23,24,25]. In the future, balloons with such devices mounted on the CNF wall are expected to be realized.

## Data Availability

Not applicable.
